# Multi-Omic Biomarkers Improve Indeterminate Pulmonary Nodule Malignancy Risk Assessment

**DOI:** 10.3390/cancers15133418

**Published:** 2023-06-29

**Authors:** Kristin J. Lastwika, Wei Wu, Yuzheng Zhang, Ningxin Ma, Mladen Zečević, Sudhakar N. J. Pipavath, Timothy W. Randolph, A. McGarry Houghton, Viswam S. Nair, Paul D. Lampe, Paul E. Kinahan

**Affiliations:** 1 Clinical Research Division, Fred Hutchinson Cancer Center, Seattle, WA 98109, USA; klastwik@fredhutch.org (K.J.L.); nma2@fredhutch.org (N.M.); houghton@fredhutch.org (A.M.H.); vnair@fredhutch.org (V.S.N.); 2 Translational Research Program, Public Health Sciences Fred Hutchinson Cancer Center, Seattle, WA 98109, USA; 3 Department of Radiology, University of Washington School of Medicine, Seattle, WA 98109, USA; vivi2011@uw.edu (W.W.); mladenze@uw.edu (M.Z.); snjp@uw.edu (S.N.J.P.); 4 Program in Biostatistics and Biomathematics, Division of Public Health Sciences, Fred Hutchinson Cancer Center, Seattle, WA 98109, USA; yzhang@fredhutch.org (Y.Z.); trandolp@fredhutch.org (T.W.R.); 5 Division of Pulmonary, Critical Care & Sleep Medicine, University of Washington School of Medicine, Seattle, WA 98195, USA; 6 Human Biology Division, Fred Hutchinson Cancer Center, Seattle, WA 98109, USA

**Keywords:** indeterminate pulmonary nodules, lung cancer, biomarkers, radiomics, semantic features, autoantibodies, glycomics, proteomics

## Abstract

**Simple Summary:**

Indeterminate pulmonary nodules detected by computer tomography are a common clinical finding, but the path to determine malignancy can cause harm to patients. We aimed to comprehensively assess other types of noninvasive information that could help clinicians diagnose lung cancer, including semantic imaging features, quantitative radiomic imaging features and proteomic, glycomic, and autoantibody–antigen complex blood-based biomarkers. Utilizing these data, we generated a malignancy risk prediction model called PSR (plasma, semantic, radiomic) comprising nine imaging and molecular biomarkers. The PSR model performed well in two cohorts and against a clinical risk prediction model. Adding known clinical risk factors for lung cancer further improved the PSR model, indicating that our discovered markers held independent clinical utility. Our study revealed novel biomarkers and a risk prediction model to help assess cancer risk in patients with indeterminate pulmonary nodules.

**Abstract:**

The clinical management of patients with indeterminate pulmonary nodules is associated with unintended harm to patients and better methods are required to more precisely quantify lung cancer risk in this group. Here, we combine multiple noninvasive approaches to more accurately identify lung cancer in indeterminate pulmonary nodules. We analyzed 94 quantitative radiomic imaging features and 41 qualitative semantic imaging variables with molecular biomarkers from blood derived from an antibody-based microarray platform that determines protein, cancer-specific glycan, and autoantibody–antigen complex content with high sensitivity. From these datasets, we created a PSR (plasma, semantic, radiomic) risk prediction model comprising nine blood-based and imaging biomarkers with an area under the receiver operating curve (AUROC) of 0.964 that when tested in a second, independent cohort yielded an AUROC of 0.846. Incorporating known clinical risk factors (age, gender, and smoking pack years) for lung cancer into the PSR model improved the AUROC to 0.897 in the second cohort and was more accurate than a well-characterized clinical risk prediction model (AUROC = 0.802). Our findings support the use of a multi-omics approach to guide the clinical management of indeterminate pulmonary nodules.

## 1. Introduction

Lung cancer is the leading cause of cancer mortality worldwide, accounting for approximately 25% of all cancer deaths [1]. Multiple clinical trials have now demonstrated at least a 20–26% reduction in cancer-related mortality through early-stage lung cancer detection with low-dose computed tomography (LD-CT) [2,3]. A recent study examining the first 1 million LD-CT screenings for lung cancer identified 170,000 patients with nodules at their baseline screen [4]. Moreover, incidental pulmonary nodules (i.e., nodules not detected during lung cancer screening) are also identified in approximately 1.5 million Americans per year [5]. Lung nodules detected by LD-CT are currently managed by assessing malignancy risk using clinical risk models [6,7,8] and management guidelines from medical societies [9,10,11,12]. These assessments are predominantly based on clinical variables and nodule characteristics with known risk factors for lung cancer like age, smoking status, and nodule size. This approach accurately assesses nodules at the polar ends of the risk spectrum where high-risk lung nodules are sent for biopsy or surgical resection, and low-risk nodules are monitored with repeat imaging. A large fraction of detected nodules, termed indeterminate pulmonary nodules (IPNs), have an intermediate risk of cancer [13] with a heterogenous clinical path. As IPNs have the highest rate of unnecessary invasive procedures and complications in patients without cancer [14], biomarkers to mitigate unnecessary risks and cost to patients are needed [15,16]. Augmenting the risk assessment of IPNs for more appropriate clinical care with noninvasive diagnostic tests is a major unmet need in clinical care.

One approach is to expand upon the information extracted from existing images using semantic features observed by radiologists or radiomic features analyzed by computer programs to reveal high-dimensional quantitative data not otherwise apparent to the human eye [17]. Radiomic features have been well documented to capture and provide rich information about shape, brightness, and texture of lesions in medical images [18]. Radiomics has the potential to uncover tumoral patterns and characteristics and provides an alternative to subjective image interpretation for improving lung cancer diagnostic accuracy [19]. Another approach is to add blood-based biomarkers. We have created a large antibody array platform to interrogate plasma sample sets and can utilize the same antibody array platform for proteomic, glycomic, and autoantibody–antigen complex interrogation by implementing three distinct probing strategies. Together, our triple hybrid platform is highly sensitive (picogram level) [20] and reproducible (coefficient of variation, CV < 10%) [20,21,22]. Using it, we identified viable proteomic biomarker candidates in ovarian [20,23,24], breast [25], pancreas [26,27], colon [21,28], and lung cancer [29,30]. Our goal for this study was to combine a multi-omics approach to noninvasively assess the malignancy risk of IPNs with computationally derived and semantic imaging features, and proteins detected by a large-scale microarray analysis of plasma.

## 2. Materials and Methods

### 2.1. Study Cohorts

**Figure 1:**
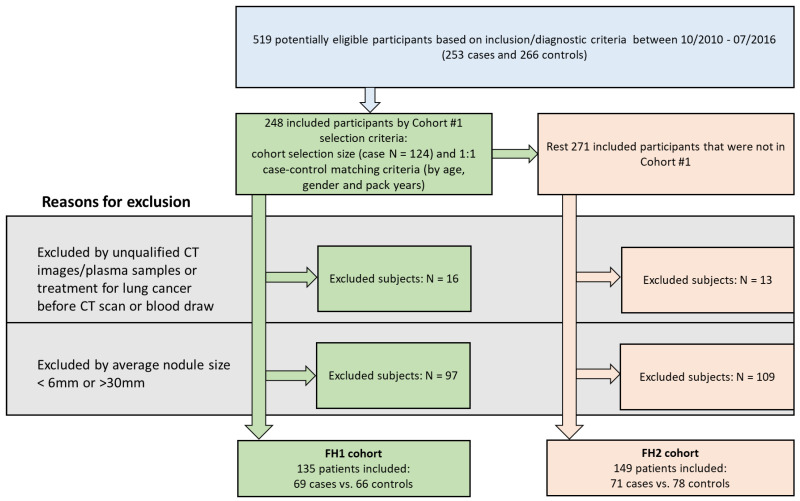
Study flow of participants. A total of 519 qualified subjects were enrolled. A total of 248 were included in Fred Hutch (FH) 1 cohort by selecting 124 cases and case–control matching 1:1 on age, gender, and pack years. The remaining 271 were evaluated for inclusion in FH2 cohort. In FH1, 113 were excluded due to unqualified CT/plasma or CT/plasma prior to treatment or nodule sizes, leaving 135 total subjects in FH1 with indeterminate pulmonary nodules. In FH2, 149 patients were included after excluding 122 subjects.

Subjects in this study were prospectively enrolled in the Fred Hutchinson (FH) Lung Cancer Early Detection and Prevention Clinic (LCEDPC) under an active institutional review board protocol from October 2010 to July 2016. Patient consent was obtained. Inclusion criteria were the following: (1) Patients incidentally found to have at least one pulmonary nodule on their CT image. (2) Patients who had both prospectively collected plasma and available CT scan images at the time of initial nodule detection. (3) For patients with malignant tumors, only subjects with nodules that were determined to be non-small-cell lung cancer (NSCLC) by histopathology were included. (4) For patients with benign nodules, only subjects with benign disease confirmed by histopathology, or resolved or stabilized nodules under CT surveillance for at least 2 years were included. The potential eligible subjects based on our inclusion/diagnostic criteria were divided into two cohorts: FH1 and FH2. Figure 1 summarizes the flowchart of the study enrolling process and reasons for patient exclusion. For the FH1 cohort, we matched 124 cases from October 2010 to May 2014 with a 1:1 controlled sample from all the potential eligible subjects by age (±2 yr), gender (exact match), and pack years (as best we could for the limited sample size). Exclusion criteria were then applied and included (1) subjects who did not have qualifying complete chest CT scans or plasma samples; (2) subjects who had treatment for lung cancer before their CT scan or blood draw; and (3) subjects with average nodule size of less than 6 mm or more than 30 mm (i.e., nodules smaller or larger than IPNs, as defined by nodules 6–30 mm, were excluded). After exclusion criteria, the FH1 cohort included 69 subjects with confirmed NSCLC (case group) and 66 with benign nodules (control group), while the FH2 cohort contained 71 cases of NSCLC and 78 controls. Clinical characteristics including age, gender, race, BMI, smoking behavior, pack years, years since quitting smoking, prior cancer history, family history of lung cancer, histology, and lung cancer stage were extracted from the electronic medical records.

### 2.2. Multidimensional Array Analysis of Plasma Samples

All laboratory steps were blind to case–control and CT scan status. Samples were analyzed on custom antibody arrays printed on Schott Nexterion slide H slides (Schott North America, Inc., Rye Brook, NY, USA) that after incubation were scanned on an Innoscan 1100 AS (Innopsys, Chicago, IL, USA) and analyzed using GenePix Pro 6.1 software (Molecular Devices, San Jose, CA, USA). After analyzing data from the FH1 cohort, for each blood-based biomarker category, we prioritized testing a subset of discovered candidates based on marker performance, antibody availability, and array space in the FH2 cohort. Antibodies printed on arrays not previously described (autoantibody array for FH1 and all arrays for FH2) are listed in Table S1.

### 2.3. Protein Analysis

As previously described [31], we removed the two most abundant proteins (albumin and IgG) using a ProtIA spin column and 200 µg of the remaining proteins from either case or control sample was labeled with NHS-Cy5. A pool of plasma from 7 healthy individuals was similarly treated and labeled with NHS-Cy3. Each individual NHS-Cy5 sample was incubated with the NHS-Cy3 pool at a 1:1 concentration on an array slide.

### 2.4. Sialyl Lewis-X- and -A-Modified Protein Analysis

As previously described [22], we detected sLeX- or sLeA-modified proteins on an antibody array using plasma diluted 1:8 in 0.05% Tween 20 in PBS. After washing, captured proteins were simultaneously probed for sLeX or sLeA modifications with Cy3-dye labeled anti-SleX (US biological, Swampscott, MA, USA) and Cy5-dye labeled anti-SleA (US biological) in FH1, or SeTau647-dye labeled anti-SleA (Fitzgerald antibodies) in FH2 (due to the discontinuation of the sLeA antibody from US Biological).

### 2.5. Autoantibody–Antigen Complex Analysis

The detection of autoantibody–antigen complexes has been previously described [21]. Patient plasma was diluted 1:80 and simultaneously probed with anti-human IgG-SeaTau647 and IgM-DyLight550 detection antibodies. Antibody array data contain a format identical to two-channel gene expression arrays and analysis proceeded analogously as described previously [21,22,31]. We expressed the differences between malignant and benign samples as a log2 odds ratio, such that a positive odds ratio means higher malignancy and negative means lower malignancy compared to benign.

### 2.6. Semantic and Quantitative Imaging Feature Analysis

The CT scans used in this study were from multiple locations within the Fred Hutchinson Cancer Center and University of Washington Medical Center from several models of multi-row spiral CT scans from GE (Boston, MA, USA), Siemens (Munich, Germany), Canon (Tokyo, Japan) (formerly Toshiba (Tokyo, Japan)), and Philips (Amsterdam, The Netherlands) scanners. The occurrence of the CT parameters in both FH1 and FH2 cohorts are presented in Table S2. An experienced thoracic radiologist (S.N.J.P.) who was blinded to clinical and histologic findings reviewed the CT images for all the included subjects. A full list of semantic features, including nodule location, nodule margin, nodule density, nodule size including long-axis diameters (*L*), short-axis diameters (*S*), height (*H*), bounding volume maximum length (VM, defined as *VM =* $\sqrt{L^{2}+S^{2}+H^{2}\;}$), number of nodules, emphysema (emphysema score, pattern, and region), fibrosis, asbestosis, invasion, and lymphadenopathy, was generated and they were qualitatively evaluated by the experienced radiologist (S.N.J.P). A second experienced thoracic radiologist (W.W.) manually delineated the trans-axial CT images slice by slice using the MIM image viewing and analysis software MIM v7.1.2 (MIM software Inc., Cleveland, OH, USA). A 3D volume of interest (VOI) was created to encompass the entire nodule and then converted to a binary mask for radiomics analysis. A total of 851 shape-, histogram-, texture- and wavelet-based features were computed using the PyRadiomics v3.0.1 (Computational imaging and bioinformatics lab at Harvard Medical School, Boston, MA, USA) for each patient [32]. A PyRadiomics feature selection workflow (Supplementary Materials) was performed to select radiomics features with good repeatability, reproducibility, and information content and less redundancy. Based on our workflow, 94 out of 851 radiomics features that exhibited the highest level of information, repeatability, and reproducibility with least redundancy were selected (Table S3).

### 2.7. Model and Statistical Analysis

Continuous variables are presented as median (range) and categorical features are presented as counts (percentages). Patient demographics between case and control groups were compared using Fisher’s Exact test for categorical variables or a Mann–Whitney U test for continuous variables. A combination of the plasma biomarkers, semantic features, and radiomics features that were described above were input into an L1-penalized (LASSO) logistic regression [33] to select a sparse marker combination using the FH1 cohort. While L1-penalized regressions are helpful for variable selection, the coefficient estimation from such models tends to bias towards zero when a large degree of regularization is employed to identify the sparse model. Therefore, in order to obtain good coefficient estimation, we further fit an L2-penalized (ridge) logistic regression model [34] to estimate the final model coefficients. Compared to the ordinary logistic regression model, the L2-penalized version provides a more optimal bias–variance tradeoff and usually improves the prediction error. The resulting plasma biomarker-, semantic-, and radiomics-based (PSR) model was further tested in an independent cohort dataset (FH2). To assess the incremental value added from demographic variables, we refit the ridge regression using the variables and the coefficients from the PSR model with age, gender, and packs of cigarettes per year added. We also applied a validated clinical risk model [6] on FH2 data to derive a Mayo prediction score. A receiver operating characteristic (ROC) curve was generated for our PSR model and the Mayo model, and the area under the curve (AUC) was calculated to quantify the predictive ability of each model. Delong test was conducted to compare the overall AUCs, and bootstrapping test was used to compare partial AUCs at 95% specificity. We also conducted calibration analysis to evaluate the prediction probability vs. the actual probability for the PSR model.

To evaluate the robustness of our marker selection, and especially the stability of the Lasso–ridge prediction modeling-building process, we combined both FH1 and FH2 data and performed 5-fold cross validation, randomly splitting the combined data into 80% training and 20% testing sets. In each training set, Lasso selected the marker combination, and the ridge model estimated the coefficients; the model was applied in testing and the tested AUC was calculated; and the procedure was repeated 100 times. An average AUC for testing set and 95% confidence interval were reported. All the analyses were performed using R statistical computing language (v. 4.1.1; R Foundation for Statistical Computing, Vienna, Austria).

## 3. Results

### 3.1. Patient Demographics and Clinical Characteristics

From 519 potentially eligible participants available from the LCEDPC, we derived two cohort sizes of 69 cases and 66 controls in FH1 and 71 cases and 78 controls in FH2 (Figure 1). As expected from an initially matched cohort, no difference was found for patient demographics and clinical characteristics between the case and control groups in FH1 except for pack years (average 25 years in controls and 33 years in cases, *p* < 0.04) (Table 1). In the unmatched FH2 cohort, patients with cancer were significantly older with a median age of 63 years (range: 33 to 87 years) in controls and a median age of 68 years (range: 48 to 94 years) in cases (*p* < 0.001) and had a higher smoking rate (58.97% current or former smokers in controls and 81.69% in cases, *p* < 0.01), with more pack years (a median pack years of 7.42 (range: 0 to 94)) in controls and a median pack years of 30 (range: 0 to 180) in cases (*p* < 0.001).Compared to the FH1 cohort, there were more never-smokers, fewer patients with prior cancer history, and fewer patients with stage III–IV NSCLC in the FH2 cohort (Table S4).

### 3.2. Specific Semantic Imaging Features and Upregulated Molecular Biomarkers Are Associated with Malignant IPNs

The descriptive univariate analysis of semantic features evaluated in both cohorts demonstrated that larger nodule size, spiculated margin, part-solid density, higher emphysema score, and presence of invasion were associated with malignancy, while smooth margin and solid density were associated with benign disease in both cohorts (Table S5).

**Figure 2:**
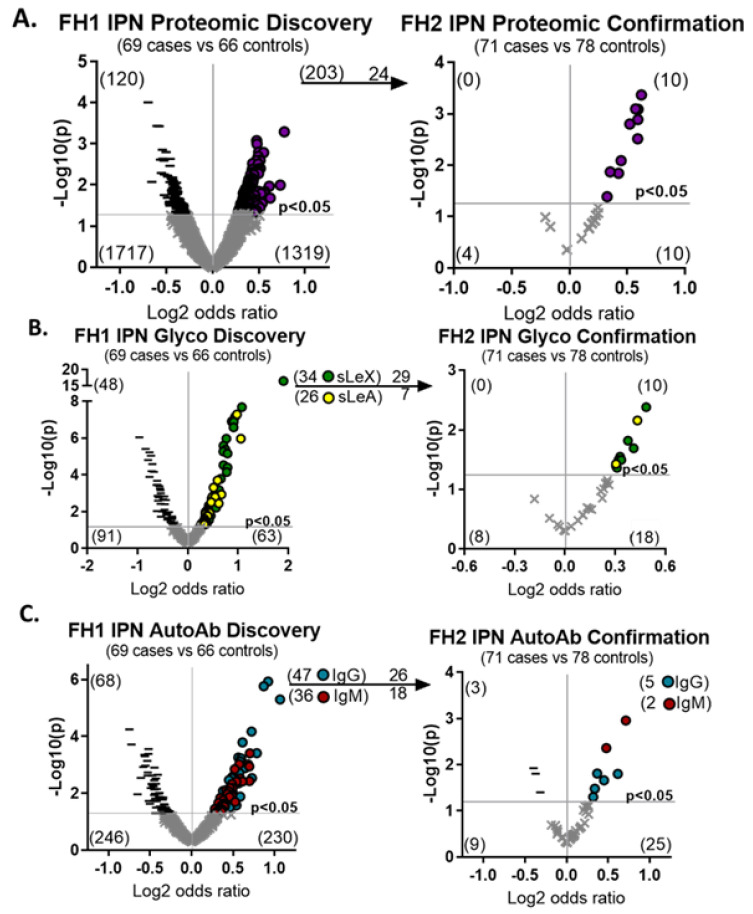
A set of hybrid plasma biomarkers are upregulated in two cohorts. Plasma biomarkers were sequentially discovered in the FH1 cohort and confirmed in FH2 and included (**A**) proteins, (**B**) sialyl Lewis glycan modifications or (**C**) autoantibody–antigen complexes. Increased expression of biomarkers in cases compared to controls are denoted by purple circles for protein, green circles for siayl Lewis X (sLeX), yellow circles for siayl Lewis A (sLeA), blue circles for IgG, and red circles for IgM. Decreased expression of biomarkers in cases compared to controls are denoted by black dashes and insignificant changes are denoted by grey ‘x’ marks.

Two-hundred and three proteins were higher in malignant IPNs at a statistical cut off of *p* < 0.05 in the FH1 cohort (Figure 2A). In the FH2 cohort, we evaluated 24 of the upregulated proteins and confirmed that 10 remained upregulated (*p* < 0.05, 41.6% confirmed). From 262 proteins assayed for the cancer-associated induction of sialyl Lewis-X or -A (sLeX/A) glycan modifications, we identified 34 proteins with sLeX modifications and 26 with sLeA modifications in the FH1 cohort (*p* < 0.05) (Figure 2B). We confirmed that 8 of the 29 sLeX modifications (*p* < 0.05, 27.6% confirmation) and 2 of the 7 sLeA modifications (*p* < 0.05, 28.6% confirmation) were present in malignant IPN in the FH2 cohort. One protein, WNT5B, had both sLeX and sLeA modifications. We tested for 427 autoantibody–antigen complexes and found that 47 IgG and 36 IgM were higher in malignant IPNs compared to benign IPNs (*p* < 0.05) in the FH1 cohort (Figure 2C). In the FH2 cohort, five IgG (*p* < 0.05, 19.2% confirmation) and two IgM (*p* < 0.05, 11.1% confirmation) remained upregulated. Cumulatively, these results indicated that we had a set of molecular and imaging biomarkers that were elevated in the plasma of patients with malignant IPNs across two independent cohorts.

### 3.3. A Combination Risk Prediction Model Can Accurately Assess Indeterminate Pulmonary Nodules

**Figure 3:**
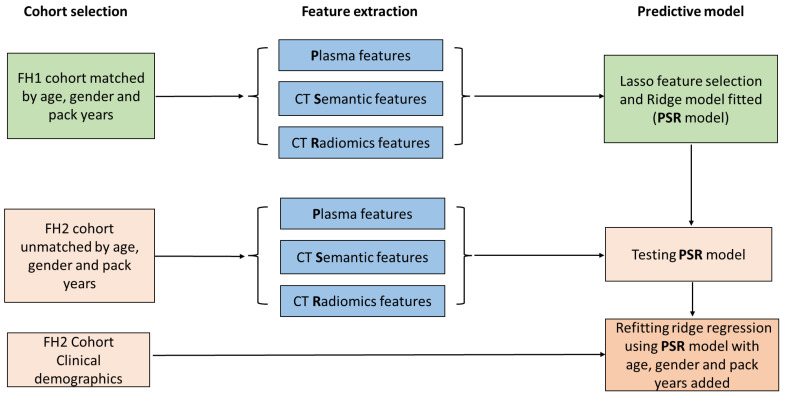
Risk prediction model study design flow chart. Subjects included in the FH1 cohort were analyzed for plasma, CT semantic, and CT radiomic features. Overall, 49 plasma, 41 CT semantic, and 94 CT radiomic features underwent Lasso feature selection and ridge model fitting to generate the risk prediction model (PSR). Subjects in the FH2 cohort also underwent multi-feature extraction and the predicted model trained in FH1 was tested in FH2. The PSR model was then refitted to add known clinical risk factors for lung cancer including age, gender, and pack years.

To find imaging and blood biomarkers that could complement each other’s performance, we used logistic regression to identify a panel of markers with the highest AUC (Figure 3). We included 49 plasma biomarkers that were upregulated (*p* < 0.1) in both cohorts (Table S6), 94 radiomic features, and 41 semantic variables for input into the penalized Lasso logistic regression model. A panel of nine biomarkers, five plasma (ALPL-sLeX, TNFRSF8-sLeX, WNT5B-sLeX, RGL1-IgG, and WNT10A-IgG), three semantic features (smooth margin, spiculated margin, and part-solid nodule density), and one radiomic feature (original_shape_LeastAxisLength) was selected. This panel of biomarkers, termed the PSR (plasma, semantic, and radiomic) model, yielded an AUC of 0.96 (95% confidence interval [CI] 0.94–0.99) in cohort 1 (Table 2). The locked PSR model in FH2 achieved an AUC of 0.85 (95% CI 0.79–0.91). An assessment of model calibration [35] showed that the model estimated risk adequately across the range of probabilities, somewhat overestimating high risk and, to a lesser extent, underestimating low risk (Supplementary Figure S1). When combining FH1 and 2 data, our PSR model had a cross-validated AUC of 0.89 (95% CI 0.87–0.92).

**Figure 4:**
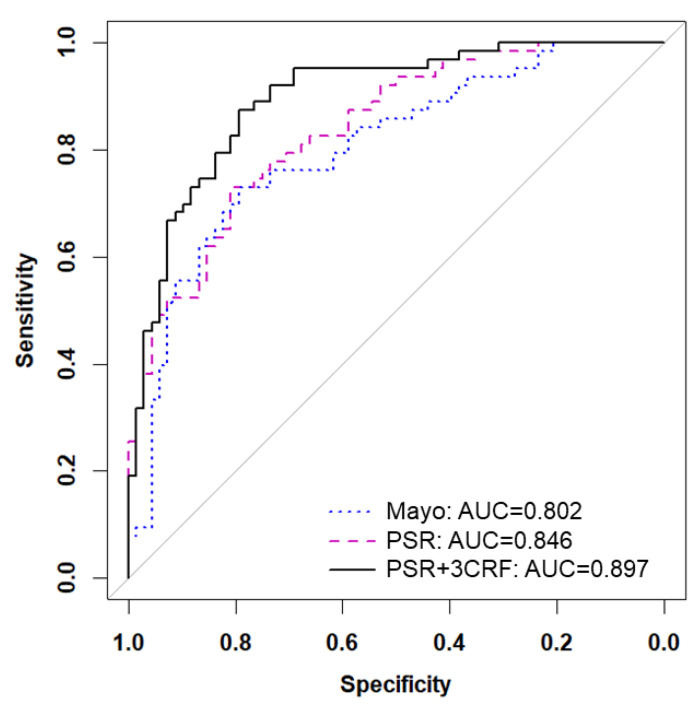
Adding known clinical risk factors improves PSR model. Receiver operating characteristic (ROC) curves are shown for the Mayo Clinic model (blue dotted line), the 9-feature PSR model (pink dashed line), and the combination of PSR and 3 clinical risk factors (3CRF) including age, gender, and pack years (black solid line).

Next, we directly compared our PSR model to the Mayo Clinic prediction model. Since 18 patients in the FH2 cohort did not contain all clinical variables involved in the Mayo model, we removed those patients and retested the fixed PSR model, which yielded a similar AUC of 0.846 (Figure 4). Applying the Mayo Clinic prediction model to this cohort yielded an AUC of 0.802. No significant difference was found overall between the tested AUCs of the PSR model and the Mayo Clinic model. However, the partial AUC at 95% specificity of the PSR model was better than the Mayo Clinic model (*p* = 0.03). Since the PSR model did not include known clinical risk factors for lung cancer, we determined if these variables would have added value. After refitting the PSR model using ridge regression, the PSR model plus age, gender, and pack years (three clinical risk factors (CRF)) achieved an AUC of 0.897 (95% CI 0.85–0.95).

## 4. Discussion

Increasing evidence supports the key role that multiplexing biomarkers can play in accurately detecting early-stage cancers [36]. In this study, we provide a comprehensive proteomic, glycomic, and autoantibody blood-based biomarker evaluation paired with semantic and quantitative imaging analysis to train and test a risk prediction model specifically for IPNs. One hundred and eighty-four biomarkers served as inputs into a parsimonious PSR risk prediction model containing only nine imaging and plasma biomarkers. The PSR model underwent multiple types of statistical analyses, including cross-validation and calibration testing. While adding three clinical risk variables (age, gender, and smoking pack years) improved the accuracy of the PSR model, it had a higher AUC compared to the Mayo Clinic model (AUC = 0.846 vs. 0.802), suggesting that PSR-derived features have diagnostic value for identifying lung cancer independent of clinical risk factors.

In the PSR risk prediction model, three semantic features (smooth margin, spiculated margin, and part-solid density) and one shape-based radiomics feature (least axis length) were selected. All three semantic features are well documented to be associated with lung cancer in the literature [13,37] and have been used in lung cancer risk prediction models [6,7,8]. The radiomics feature has been reported to be highly reproducible [38,39] and has been selected in other radiomics-derived prediction models in oncology studies [40,41,42].

Most current clinical cancer biomarkers are specific for glycoproteins or carbohydrate structures and changes in these structures can have diagnostic value [43]. Additionally, autoantibodies are known to be an attractive source for lung cancer’s early detection as they reflect a tumor-specific humoral immune response [30] and can be detected months to years prior to clinical diagnosis [44]. Importantly, while proteomic markers were included in the model-building process, only glycomic and autoantibody biomarkers contributed to the final PSR model. Two secreted wingless-type (WNT) ligands, WNT5B and WNT10A, were incorporated as glycomic and autoantibody biomarkers in the PSR, respectively. The WNT family is known to play a supportive role in lung tumorigenesis [45] and WNT family inhibitors are currently in clinical trials [45,46]. Tumor necrosis family receptor superfamily 8 (TNFRSF8 or CD30) is upregulated in lung cancer and is being investigated as a target for immunoPET noninvasive imaging [47]. We demonstrate here that cancer-specific TNFRSF8 glycosylation is associated with malignant lung nodules. Recently, we reported the identification of post-translationally modified proteins targeted by autoantibodies in SCLC that distinguished tumor-specific antigens from normal tissue [29]. As RGL1 and WNT10A are widely expressed in both normal and cancer tissues [48,49], determining the immunogenic epitopes that autoantibodies target could lend insight into the interactions of the immune system and tumorigenesis.

Given the importance of lung cancer’s early detection and the reduced mortality associated with CT screening, multiple groups have proposed prediction models that contain imaging features and/or blood-based biomarkers [6,8,42,50,51,52,53]. Biomarkers from other sources are also actively being investigated and include airway epithelia from bronchial brushings [54], exhaled breath [55], sputum [56], and urine [57]. However, integrating complementary but distinct biomarkers is likely to be required to capture all patients with lung cancer. Nodify XL2 incorporates two plasma proteins and clinical risk factors to evaluate IPNs and, in a prospective observational trial, their algorithm yielded a negative predictive value of 98% at the cost of a low positive predictive value [58]. In the prospective Early Diagnosis of Lung Cancer Scotland trial, the EarlyCDT-Lung test, a blood-biomarker panel consisting of seven autoantibodies, was used to direct CT screening in high-risk patients. A positive EarlyCDT-Lung test triggered LDCT screening every 6 months and reduced the number of late-stage (III/IV, hazard ratio 0.64) lung cancers diagnosed compared to standard clinical practice [59]. The CBM model combined the blood-based biomarker Cyfra 21-1, radiomic features, and clinical characteristics to improve the diagnostic accuracy of IPNs in four independent cohorts [60]. All of these studies show promise but better performance, likely in the form of additional biomarkers, is needed for optimal clinical utility. In addition to the lung cancer risk assessment of IPNs, biomarkers also have the potential to inform multiple steps in the clinical management of lung cancer. Biomarkers can optimize risk-based selection for screening eligibility to identify at-risk individuals in populations outside of LCS [61]. For example, a four-marker protein panel of blood-based biomarkers in combination with a clinical risk prediction model of patient characteristics more accurately identified persons who would benefit from lung cancer screening compared with the US Preventive Services Task Force screening criteria [51]. Noninvasive biomarkers can also aid in assessing patient prognosis, treatment eligibility, and therapeutic response [62]. Despite the potential, no risk prediction model utilizing biomarkers has reached widespread clinical implementation.

This study has limitations. It was a retrospective dataset from one center that relied on banked plasma samples and may result in certain biases. Moving forward, it will be key to validate the PSR risk prediction model in cohorts from outside institutions to confirm the findings reported here. These studies are currently ongoing. Another limitation pertains to the manual delineation of the nodules. While manual segmentation is widely regarded as the golden standard in imaging analysis, it is a time-consuming process and not practical for routine clinical practice. Furthermore, both the semantic and radiomics analyses relying on manual segmentation are susceptible to intra- and interobserver variability. Future directions involve the implementation of an auto-segmented algorithm for imaging analysis and the utilization of deep learning methods, wherein the entire CT scan can be employed as input. Ultimately, we envision assigning a high or low risk score to each IPN patient using our risk prediction model. A high risk prediction score would identify patients in need of immediate diagnostic follow up. A low risk prediction score would indicate patients who could be monitored with repeat imaging.

## 5. Conclusions

In summary, our results demonstrate the promising utility of combining multiple types of noninvasive approaches to assess the lung cancer risk of IPNs. Combining hybrid biomarkers with known clinical risk factors for lung cancer further improved the accuracy of a risk prediction model. These data suggest that integrated imaging and blood-based biomarkers can beneficially augment the current risk assessments used for the clinical management of IPNs.

## Figures and Tables

**Table 1:** Participant demographics and clinical characteristics in Fred Hutch (FH) 1 and FH2 cohorts. Body mass index (BMI), non-small-cell lung carcinoma (NSCLC), adenocarcinoma (AD), squamous cell carcinoma (SCC). * Missing values present in BMI (*n* = 1), BMI class (*n* = 1), family history of lung cancer (*n* = 3), years since quitting smoking (*n* = 2). † Fisher’s exact test (categorical) or Wilcoxon rank-sum test (continuous variables) comparing patients with and without malignancy for two FH cohorts.

Variable	FH1 (*n* = 135)		P †	FH2 (*n* = 149)		P †
	Control (*n* = 66)	Case (*n* = 69)		Control (*n* = 78)	Case (*n* = 71)	
**Gender (M)**	33 (50.0%)	31 (44.9%)	0.61	47 (60.26%)	38 (53.52%)	0.41
**Age**	67 (40–91)	67 (44–83)	0.74	63 (33–87)	68 (48–94)	<0.001
**Race**			0.37			0.72
African American	0 (0.00%)	1 (1.45%)		4 (5.13%)	1 (1.41%)	
Asian	1 (1.52%)	4 (5.80%)		3 (3.85%)	5 (7.04%)	
Caucasian	63 (95.45%)	62 (89.86%)		68 (87.18%)	62 (87.32%)	
Hispanic or Latino	0 (0.00%)	1 (1.45%)		1 (1.28%)	0 (0.00%)	
Native American	0 (0.00%)	1 (1.45%)		1 (1.28%)	1 (1.41%)	
Native Hawaiian/Pacific Islander	1 (1.52%)	0 (0.00%)		0 (0.00%)	1 (1.41%)	
Unknown/Not Reported	1 (1.52%)	0 (0.00%)		1 (1.28%)	1 (1.41%)	
**BMI** *	26.7 (18.1–58.7)	26.6 (17.9–47.4)	0.86	26.5 (18.3–52.9)	26.1 (16.1–42.8)	0.37
**BMI class** *			0.86			0.64
Normal	24 (36.92%)	28 (40.58%)		31 (39.74%)	30 (42.25%)	
Overweight	23 (35.38%)	21 (30.43%)		24 (30.77%)	25 (35.21%)	
Obese	18 (27.69%)	20 (28.99%)		23 (29.49%)	16 (22.54%)	
**Smoking status**			0.10			<0.01
Current smoker	17 (25.76%)	28 (40.58%)		15 (19.23%)	14 (19.72%)	
Former smoker	34 (51.52%)	33 (47.83%)		31 (39.74%)	44 (61.97%)	
Never smoker	15 (22.73%)	8 (11.59%)		32 (41.03%)	13 (18.31%)	
Years since quitting *	1.5 (0–58)	0 (0–53)	0.59	0 (0–64)	2 (0–52)	0.23
**Prior cancer history** (Y)	26 (39.39%)	21 (30.43%)	0.29	19 (24.36%)	14 (19.72%)	0.56
**Family history of lung cancer** * (Y)	13 (20.00%)	22 (31.88%)	0.17	25 (32.89%)	17 (23.94%)	0.27
**Histology for NSCLC**						
AD		56 (81.16%)			51 (71.83%)	
AD, SCC		0 (0.00%)			1 (1.41%)	
Large-cell carcinoma		1 (1.45%)			0 (0.00%)	
SCC		12 (17.39%)			19 (26.76%)	
**NSCLC cancer stage**						
0		2 (2.90%)			0 (0.00%)	
I		40 (57.97%)			55 (77.46%)	
II		4 (5.80%)			8 (11.27%)	
III		9 (13.04%)			4 (5.63%)	
IV		14 (20.29%)			4 (5.63%)	

**Table 2:** Plasma, semantic, and radiomic (PSR) model performance in FH1 and FH2 cohorts. The # symbol denotes number.

PSR Model	# of Subjects (6~30 mm)	# of Variables Considered	Lasso Selected	6~30 mm Nodule (AUC)
Training on FH1	69 case vs. 66 ctrl	188	9	0.964
Testing on FH2	71 case vs. 78 ctrl	9		0.846

## Data Availability

Supporting data are available from the corresponding authors.

## Supplementary Materials

The following supporting information can be downloaded at https://www.mdpi.com/article/10.3390/cancers15133418/s1: Supplementary methods of five-step radiomic feature selection process [63,64,65]; Table S1: Antibodies printed on microarrays; Table S2: Distribution of CT parameters in FH cohorts 1 and 2; Table S3: Selected 94 reproducible radiomic features input into Lasso selection; Table S4: Comparison of demographics between FH cohorts 1 and 2; Table S5: Distribution of semantic features in cohorts FH1 and 2; Table S6: Plasma biomarker input into Lasso selection; Figure S1: Calibration of the PSR model in FH2 cohort.
