# Supplementary material for: Multi-Omic Biomarkers Improve Indeterminate Pulmonary Nodule Malignancy Risk Assessment
Source: Cancers (Basel). 2023 Jun 29;15(13):3418. doi: 10.3390/cancers15133418 (PMC10341085; doi:10.3390/cancers15133418)
Supplement: Supplementary file 1 [file cancers-15-03418-s001.zip › cancers-2414747-supplementary.pdf]

Supplemental Materials

Supplemental Methods:

To enhance the reproducibility of radiomics analysis, we developed a 5-step feature selection process: 1) repeatability analysis using RIDER lung CT dataset[65], only the features with intraclass correlation coefficient (ICC) greater than 0.85 were retained. 2) reproducibility analysis on multiple observers using NSCLC-Radiomics-Interobserver1 dataset[66], only the features with ICC greater than 0.75 were retained. 3) reproducibility analysis on convolution kernels using an internal subset (N=86) of CT images with paired soft and sharp convolution-kernel reconstructions. We smoothed the images from sharp kernels first and compared them to the images from soft kernels. Only the features with ICC greater than 0.90 were retained. 4) information content analysis using a cohort with CT images of pulmonary nodules in all sizes (N=496) and the method described in Du D et al[67]. 5) redundancy analysis using the same cohort in 4). Of every two features with Pearson correlation < 0.90, the feature with the larger mean ICC was retained. After the feature selection, 94 out of 851 radiomics features that exhibited the highest level of information, repeatability and reproducibility with least redundancy were selected.

Table S1. List of antibodies printed on arrays.

Antibody Name	Supplier	Cat #	Array Type
EpCAM	SDI	1931.00.02	Auto
CP	SDI	1879.00.02	Auto
CLEC3B	SDI	1875.00.02	Auto
ADRB2	SDI	1956.00.02	Auto
OWL	One World Labs	500-P227	Auto
CEACAM5	SDI	1667.00.02	Auto
CTSE	Abnova	H00001510-B01P	Auto
MUC5AC	Abnova	H00004586-M07	Auto
KRT10	Abnova	H00003858-M01	Auto
GLI3	Abnova	H00002737-M01	Auto
CD44	SDI	4084.00.02	Auto
CREBBP	Aviva	ARP43609_P050	Auto
SFTPB	Aviva	ARP41411_P050	Auto
CTTN	Aviva	ARP52067_P050	Auto
ST6GALNAC1	Aviva	ARP47446_P050	Auto
ST3GAL1	Aviva	ARP45410_P050	Auto
ALDOC	Aviva	ARP48274_P050	Auto
SIGLEC6	Aviva	ARP41925_P050	Auto
SELE	Aviva	ARP59109_P050	Auto
CCND1	Santa Cruz Biotechnology	sc-718	Auto
NEU3	SDI	2163.00.02	Auto
ASH2L	SDI	2129.00.02	Auto
XBP1	SDI	2054.00.02	Auto
CD46	SDI	2050.00.02	Auto
TET1	SDI	2126.00.02	Auto

EN1	SDI	2104.00.02	Auto
CTPS	SDI	2337.00.02	Auto
WNT4	SDI	2299.00.02	Auto
TPM3	SDI	2272.00.02	Auto
IFNg	SDI	2335.00.02	Auto
VHL	SDI	2319.00.02	Auto
UBE2S	OWL	AP2007lb	Auto
KDM5B	SDI	2226.00.02	Auto
PW-1L (CCSP-1)	CCSP-1 Antibody	PW-1L	Auto
TCEB3	Abcam	AB88087	Auto
NTRK1-3	Abcam	AB89925	Auto
COL18A1	LSBio	LS-C104551	Auto
THBS1	LSBio	LS-B2570	Auto
C4B	Abcam	AB54902	Auto
COL5A3	Santa Cruz Biotechnology	sc-240246	Auto
SACS	SDI	2434.00.02	Auto
MBL2	SDI	2445.00.02	Auto
PRCC	SDI	2518.00.02	Auto
Novus	Novus	NBP2-14835	Auto
SUFU	SDI	2487.00.02	Auto
BSG	SDI	2602.00.02	Auto
MUC16	SDI	2545.00.02	Auto
KLK13	SDI	2633.00.02	Auto
PLG	SDI	2640.00.02	Auto
ESR2	SDI	2625.00.02	Auto
AFP	SDI	2544.00.02	Auto
CD44	Abcam	ab41478	Auto
XRCC3	SDI	2728.00.02	Auto
CXADR	SDI	2674.00.02	Auto
EPHA2	SDI	2662.00.02	Auto
EGF	SDI	2661.00.02	Auto
IER3	SDI	2827.00.02	Auto
CASP8	SDI	2802.00.02	Auto
EVI1	SDI	2810.00.02	Auto
JMJD1C	SDI	2983.00.02	Auto
DST	SDI	2972.00.02	Auto
EGFR	SDI	3177.00.02	Auto
IL7	SDI	3340.00.02	Auto
F13A1	SDI	3319.00.02	Auto
CSF2	SDI	3309.00.02	Auto
ALPL	SDI	3292.00.02	Auto

SCN3B	SDI	3747.00.02	Auto
OWL	One World Labs	20278	Auto
BCL3	Santa Cruz Biotechnology	sc-13038	Auto
SPDYA	SDI	4425.00.02	Auto
SUZ12	SDI	4616.00.02	Auto
SLC30A8	Origene	TA590368	Auto
SLC30A8	LSBio	LS-C296475	Auto
ARGHDIB	One World Labs	EB10223	Auto
IL13	R&D systems	MAB2131	Auto
TFF3	R&D systems	MAB4408	Auto
TNFAIP6	R&D systems	MAB2104	Auto
FETUB	R&D systems	MAB17251	Auto
CD44	SDI	2806.00.02	Auto
INHA	SDI	3342.00.02	Auto
YES1	SDI	2692.00.02	Auto
PHB	SDI	2684.00.02	Auto
RECQL4	SDI	2547.00.02	Auto
WNT5B	SDI	2301.00.02	Auto
EPHX1	Abnova	H00002052-B01P	Auto
SMARCA2	Abcam	ab15597	Auto
CA2	Abcam	AB6621-5	Auto
HIST1H1D	Abcam	ab24174	Auto
HLF	Abnova	H00003131-M01	Auto
ILK	Sigma	I1907	Auto
VWF	Sigma	F3520	Auto
HP	Sigma	H8636-1vl	Auto
BMF	Sigma	B1684-100ug	Auto
AOAH	SDI	4572.00.02	Auto
ENO2	SDI	3318.00.02	Auto
SIN3A	Santa Cruz Biotechnology	SC-994	Auto
ADRB2	SDI	1955.00.02	Auto
CLSTN2	SDI	3582.00.02	Auto
ITGA6	SDI	2465.00.02	Auto
ABL2	SDI	2327.00.02	Auto
REC8	SDI	2947.00.02	Auto
WNT7A	SDI	2303.00.02	Auto
UTP20	SDI	1691.00.02	Auto
SPINK2	SDI	3257.00.02	Auto
IL2	SDI	2041.00.02	Auto
VWF	SDI	3270.00.02	Auto
RGL1	SDI	3826.00.02	Auto

SELENBP1	SDI	2643.00.02	Auto
CCL14	Abcam	AB9845	Auto
IGF1R	SDI	2384.00.02	Auto
CD33	SDI	1918.00.02	Auto
FZD2	Aviva	ARP41236_P050	Auto
CCR5	SDI	1927.00.02	Auto
XRCC5	SDI	2730.00.02	Auto
DDHD2	SDI	2140.00.02	Auto
CDH20	SDI	3574.00.02	Auto
GAPDH	Aviva	ARP40175_P050	Auto
LIN61	SDI	2959.00.02	Auto
CALD1	SDI	2238.00.02	Auto
CCR2	SDI	1945.00.02	Auto
BTK	SDI	2656.00.02	Auto
IFNB1	SDI	2459.00.02	Auto
JAZf1	SDI	2716.00.02	Auto
ELP2	SDI	3087.00.02	Auto
NOV	SDI	3386.00.02	Auto
PSAT1	Aviva	ARP46303_P050	Auto
USH1c	SDI	2092.00.02	Auto
GAK1	SDI	2946.00.02	Auto
RPS6KA2 (S380)	Cell Signaling	9335	Auto
IMMP1L	SDI	1729.00.02	Auto
CCL20	Abcam	AB89395	Auto
CHUK (S176/180)	Cell Signaling	2697	Auto
WNT3A	Cell Signaling	2721	Auto
RBL2	Abcam	AB89459	Auto
MAPK1/3 (T202/Y204)	Cell Signaling	4370	Auto
LMN1	SDI	3853.00.02	Auto
FCN2	SDI	2446.00.02	Auto
PIK3CD	R&D systems	MAB2687	Auto
FOXO1	SDI	2558.00.02	Auto
ERCC2	SDI	2585.00.02	Auto
WNK2	SDI	2591.00.02	Auto
EFNB3	Abcam	AB89474	Auto
hda-1	SDI	3866.00.02	Auto
SPFH2	SDI	2132.00.02	Auto
LPXN	SDI	4391.00.02	Auto
TMPRSS11E	SDI	4546.00.02	Auto
HSPA5	SDI	2822.00.02	Auto
SPINK1	SDI	3475.00.02	Auto

DAPK1	SDI	2848.00.02	Auto
TNC	SDI	4170.00.02	Auto
HOXD13	Aviva	OAAB07467	Auto
DCP1B	Aviva	OAAB06487	Auto
CXCL5	SDI	3314.00.02	Auto
FSTL1	SDI	3141.00.02	Auto
CSTF1	SDI	2715.00.02	Auto
IL12RB2	SDI	2981.00.02	Auto
GPR125	SDI	2902.00.02	Auto
ANGPTL4	SDI	2620.00.02	Auto
FBXW7	SDI	3049.00.02	Auto
PHLPPL	SDI	3818.00.02	Auto
CTSD	SDI	2258.00.02	Auto
CDH23	SDI	2094.00.02	Auto
SERPIND1	R&D systems	MAB3198	Auto
IL34	R&D systems	MAB52651	Auto
IL1B	R&D systems	MAB601	Auto
CXCL12	R&D systems	MAB350	Auto
PCSK9	R&D systems	MAB28881	Auto
COL4A3	Santa Cruz Biotechnology	sc-18178	Auto
MGST1	Santa Cruz Biotechnology	sc-17003	Auto
SDC1	Santa Cruz Biotechnology	sc-7099	Auto
CBLC	Santa Cruz Biotechnology	sc-8371	Auto
IL28A	R&D systems	MAB15871	Auto
TLR9	Santa Cruz Biotechnology	SC-47723	Auto
HIF1A	Santa Cruz Biotechnology	SC-13515	Auto
ERBB4	R&D systems	MAB11311	Auto
MAPK11	R&D systems	MAB5885	Auto
EGFR	Santa Cruz Biotechnology	sc-003	Auto
GPANK1 (BAT4)	Santa Cruz Biotechnology	SC-79861	Auto
MAP2K4	R&D systems	MAB3390	Auto
PTK2	Santa Cruz Biotechnology	sc-558G	Auto
ENO2	R&D systems	AF5169	Auto
CDK1(Y15)	R&D systems	AF888	Auto
IL15	R&D systems	MAB247	Auto
PW-4H (CCSP-1)	CCSP-1 antibody	PW-4H	Auto
IP-5.1H (CCSP-1)	CCSP-1 antibody	IP-5.1H	Auto
4-5F1 (CCSP-2)	CCSP-2 antibody	4-5F1	Auto
3-2E5 (CCSP-2)	CCSP-2 antibody	3-2E5	Auto
ATF3	Santa Cruz Biotechnology	SC-188	Auto
MYC	Santa Cruz Biotechnology	SC-788	Auto

CDCP1	R&D systems	MAB26661	Auto
SPRED1	R&D systems	AF5067	Auto
IL17D	R&D systems	MAB1504	Auto
MAP2K4 (S257/T261)	R&D systems	AF2990	Auto
MDH2	Aviva	ARP48286_T100	Auto
CXCR3	SDI	2613.00.02	Auto
AXL	Aviva	ARP59004_P050	Auto
HOXC11	Aviva	ARP31960_P050	Auto
FANCC	SDI	2529.00.02	Auto
BUB1B	SDI	2373.00.02	Auto
WISP1	SDI	2320.00.02	Auto
THBS2	SDI	2442.00.02	Auto
GDF15	Aviva	ARP42200_P050	Auto
KRT10	Aviva	ARP41730_P050	Auto
CDC25C	SDI	2908.00.02	Auto
MGAT3	Abnova	H00004248-M01	Auto
TBC1D3	SDI	2080.00.02	Auto
CEACAM1	SDI	2037.00.02	Auto
TNFRSF10A	SDI	2646.00.02	Auto
C4orf7	SDI	2062.00.02	Auto
MMP16	SDI	2266.00.02	Auto
TRPV6	SDI	4347.00.02	Auto
CHUK (S176)/IKBKB (S177)	Cell Signaling	2078	Auto
FH	SDI	2488.00.02	Auto
PGF	SDI	2624.00.02	Auto
MMEL1	SDI	3215.00.02	Auto
CLNS1A	SDI	2657.00.02	Auto
ULBP2	SDI	2708.00.02	Auto
BTN3A2	SDI	2801.00.02	Auto
WNT10A	SDI	2309.00.02	Auto
ATP6AP1	SDI	2229.00.02	Auto
RAB6B	SDI	2432.00.02	Auto
XIAP	SDI	2995.00.02	Auto
TIE1	SDI	2704.00.02	Auto
ITGAV	SDI	2466.00.02	Auto
CD276	SDI	2622.00.02	Auto
IL1B	SDI	2360.00.02	Auto
PTGS1	SDI	2430.00.02	Auto
SERPINB5	LSBio	LS-C104541	Auto
WASL	Abnova	H00008976-M04	Auto
TSP50	SDI	1719.00.02	Auto

MES3	SDI	3858.00.02	Auto
Lcp2	SDI	2985.00.02	Auto
NOTCH1	SDI	2273.00.02	Auto
XPC	SDI	2551.00.02	Auto
MES2	SDI	3857.00.02	Auto
CLGN	SDI	3127.00.02	Auto
IL4R	SDI	1968.00.02	Auto
ENO1	Abcam	ab35075	Auto
LMTK2	SDI	3758.00.02	Auto
IL19	SDI	2447.00.02	Auto
MYST3	SDI	2162.00.02	Auto
SFN	SDI	2435.00.02	Auto
NCOA6	SDI	3683.00.02	Auto
DEPDC1	SDI	2663.00.02	Auto
METTL3	GenWay	18-003-42560	Auto
TRAF4	SDI	1717.00.02	Auto
NRP1	SDI	3037.00.02	Auto
ACADM	Novus	NB 100-1121	Auto
MUC2 (SMUC)	Abcam	AB22712	Auto
RALA	BD Transduction	610221	Auto
SERPINB6	Novus	NB100-93464	Auto
PRG2	Abcam	AB20291-100	Auto
MARCO	Abcam	ab33453	Auto
ITGB1	Abcam	AB7168	Auto
IFNG	Abcam	ab13783	Auto
HIF1A	Santa Cruz Biotechnology	SC-12542	Auto
TNFRSF1B	Abcam	AB10503	Auto
POLG2	SDI	2170.00.02	Auto
EPHA4	Abnova	H00002043-M03	Auto
MMP11	SDI	1980.00.02	Auto
SKI	SDI	2755.00.02	Auto
STAT1	SDI	2697.00.02	Auto
PSCD3	SDI	2057.00.02	Auto
TNFRSF9	Abcam	AB89085	Auto
FBXW7	SDI	3050.00.02	Auto
LALBA	SDI	3353.00.02	Auto
MET	Santa Cruz Biotechnology	sc-161	Auto
ARFIP2	Abcam	AB56988	Auto
ERBB3	SDI	2259.00.02	Auto
SCN9a	SDI	2056.00.02	Auto
PARK7	Sigma	D2943	Auto

HDAC2	SDI	2821.00.02	Auto
C10orf10	SDI	2083.00.02	Auto
AR	SDI	2215.00.02	Auto
AAMP	SDI	2443.00.02	Auto
AQP2	SDI	1962.00.02	Auto
IL20(std)	R&D systems	MAB11027	Auto
SMAD4	Santa Cruz Biotechnology	sc-7966x	Auto
TNFRSF1B	Abcam	ab17038	Auto
TARBP1	SDI	3072.00.02	Auto
BRF2	SDI	2713.00.02	Auto
CCNB2	SDI	2900.00.02	Auto
TGIF1	SDI	3059.00.02	Auto
XRCC1	SDI	2727.00.02	Auto
LHX1	SDI	2152.00.02	Auto
FOLR3	SDI	2148.00.02	Auto
SETD5	SDI	2256.00.02	Auto
RPS6KA5 (S212)	R&D systems	AF1036	Auto
PF4	Abcam	AB9561	Auto
RAD23A	Abcam	ab55725	Auto
MAPK7	Santa Cruz Biotechnology	Sc-5626	Auto
RAPGEF1	Santa Cruz Biotechnology	sc-869	Auto
GALK1	Abcam	AB14023	Auto
IL4	R&D systems	MAB204	Auto
CTLA4	SDI	1964.00.02	Auto
CDKN1A	Santa Cruz Biotechnology	sc-397	Auto
TSG101	Santa Cruz Biotechnology	SC-22774	Auto
CBL	Santa Cruz Biotechnology	sc-170	Auto
WB-4H (CCSP-1)	CCSP-1 antibody	WB-4H	Auto
3-1H1 (CCSP-2)	CCSP-2 antibody	3-1H1	Auto
MET	R&D systems	MAB3582	Auto
SFRP1	Abcam	ab7797	Auto
SFRP4	R&D	AF3697	Auto
A2M	R&D	AF2420	Auto
FOLR3	R&D	AF4148	Auto
CRKL	R&D	AF2964	Auto
HSPA8	R&D systems	AF3398	Auto
KRT8	R&D systems	MAB1938	Auto
INPP5D	R&D systems	MAB4356	Auto
FABP1	R&D systems	MAB3165	Auto
FKBP2	R&D systems	MAB2317	Auto
epb41l3	Aviva	ARP73951_P050	Auto

cdca7l	Aviva	ARP57292_P050	Auto
chtf18	Aviva	ARP57589_P050	Auto
fcgr2a	Aviva	OAAB10024	Auto
ints10	Aviva	ARP70587_P050	Auto
hmga1	Aviva	ARP38111_P050	Auto
lrrc45	Aviva	OAAB07613	Auto
lingo1	Aviva	OAAB04259	Auto
hif3a	Aviva	ARP39936_T100	Auto
epb41l3	Aviva	OAGA01718	Auto
rps13	Aviva	ARP56179_P050	Auto
rps13	Aviva	OAAB02220	Auto
hmga1	Aviva	OAAB05695	Auto
ankrd36b	Aviva	ARP68793_P050	Auto
lingo1	Aviva	OAAB02513	Auto
chtf18	Aviva	OAAB09272	Auto
cdca7l	Aviva	OAAB17991	Auto
hif3a	One World Labs	TA800720	Auto
ints10	One World Labs	ESAP11026	Auto
pla2g4c	One World Labs	R12-3333	Auto
hif3a	One World Labs	EB10073	Auto
s100a7l2	One World Labs	R12-3476	Auto
s100a7l2	One World Labs	ENT4202	Auto
fcgr2a	One World Labs	TA500645	Auto
pla2g4c	One World Labs	EB10114	Auto
fcgr2a	One World Labs	UM500051	Auto
cdca7l	One World Labs	TA803020	Auto
epb41l3	One World Labs	EB07024	Auto
VGCC (CACNA1A)	Aviva	oabb0146	Auto
Ta (PNMA2)	Aviva	arp52314_p050	Auto
Yo (PCA2 CDR2)	Aviva	arp51161_t100	Auto
Rc	Aviva	oaan01784	Auto
Sox	Aviva	arp31735_p050	Auto
GABA-b	R&D systems	af7000	Auto
GAD65	R&D systems	af2247	Auto
CRMP5	Novus	nbp1-33419	Auto
Hu (ELAVL2)	Aviva	oasg03682	Auto
Ma (PNMA1)	Aviva	arp33097_p050	Auto
EpCAM	SDI	1931.00.02	Auto
NTNG2	SDI	3686.00.02	Proteomic
NAV3	SDI	3682.00.02	Proteomic
FRMD3	SDI	3757.00.02	Proteomic

THBS3	SDI	3819.00.02	Proteomic
KIFC3	SDI	4661.00.02	Proteomic
NELL2	SDI	4603.00.02	Proteomic
SLC30A8	Origene	TA590368	Proteomic
SLC30A8	LSBio	LS-C296475	Proteomic
PTPRU	R&D systems	MAB7475	Proteomic
lingo1	Aviva	OAAB04259	Proteomic
CD22	PeproTech	500-P227	Proteomic
PHB	SDI	2684.00.02	Proteomic
PHLPPL	SDI	3818.00.02	Proteomic
TRPV6	SDI	4347.00.02	Proteomic
FSTL1	SDI	3141.00.02	Proteomic
hda-1	SDI	3866.00.02	Proteomic
SPFH2	SDI	2132.00.02	Proteomic
MMP16	SDI	2266.00.02	Proteomic
ERCC2	SDI	2585.00.02	Proteomic
TNFRSF1B	Abcam	AB10503	Proteomic
ARFIP2	Abcam	AB56988	Proteomic
LPXN	SDI	4391.00.02	Proteomic
s100a7l2	AssayBiotech	R12-3476	Proteomic
NCK1	SDI	2480.00.02	Proteomic
fcgr2a	Aviva	OAAB10024	Proteomic
ankrd36b	Aviva	ARP68793_P050	Proteomic
epb41l3	Aviva	ARP73951_P050	Proteomic
INHBC	Aviva	OASG03873	Proteomic
TGFB2	Aviva	OASG07155	Proteomic
CD70	Aviva	OASG01345	Proteomic
GPR19	Aviva	OASG03154	Proteomic
LST1	Aviva	ARP64242	Proteomic
RUNX3	Aviva	OASG06510	Proteomic
GADD45G	Aviva	OASG02914	Proteomic
S100A7	Aviva	OACD07110	Proteomic
INHBA	Aviva	OASG03869	Proteomic
KIFC3	ProteinTech	10125-2-AP	Proteomic
CAMK1	Aviva	ARP88196	Proteomic
NELL2	Thermo	PA5-57373	Proteomic
ATG7	Aviva	OAAJ06604	Proteomic
NAV3	Thermo	PA5-56932	Proteomic
BRCA1	Novus	NB100-598SS	Proteomic
THBS3	Aviva	OAAB14510	Proteomic
CSF3	Aviva	OAAB15063	Proteomic

EPHA2	Aviva	OAAB17127	Proteomic
TECTA	Thermo	PA5-80102	Proteomic
IL13RA1	R&D systems	MAB1462	Proteomic
CSF2	Abcam	AB14024	Proteomic
CRB3	Novus	NBP1-81185	Proteomic
RAB5A	Abcam	ab18211	Proteomic
CRB3	Novus	NBP1-98328	Proteomic
SMAD2	Novus	NB100-56462SS	Proteomic
MED12	Novus	NB100-2357	Proteomic
SMAD2	R&D systems	MAB4037-SP	Proteomic
MED12	Aviva	OASG07308	Proteomic
BECN1	BD	612112	Proteomic
MED12	Aviva	OACA09933	Proteomic
FN1	BD	610077	Proteomic
PRKCSH	Novus	NBP2-38492	Proteomic
BAX	BD	610982	Proteomic
MYO18B	Novus	NBP1-89967	Proteomic
CSF2	Abcam	ab54429	Proteomic
DNAJC10	Abnova	H00054431-M01	Proteomic
LIFR	Abcam	AB89792	Proteomic
SLC9A3R1	Abcam	ab9526-100	Proteomic
FMN1	SDI	4411.00.02	Proteomic
EXT2	SDI	2486.00.02	Proteomic
CSF3	SDI	3310.00.02	Proteomic
CDKN1A	Biolegend	626701	Proteomic
CD24	AbD Serotec	MCA1379T	Proteomic
MUC17	AbD Serotec	MCA1837	Proteomic
PDLIM1	Abcam	AB77142	Proteomic
VWF	Abcam	ab6994	Proteomic
B2M	Abcam	ab27588-100	Proteomic
PPIE	Abnova	H00010450-M02	Proteomic
CTSD	R&D systems	MAB1014	Proteomic
REG3A	R&D systems	AF5940	Proteomic
FBLN5	R&D systems	MAB3095	Proteomic
FUT3	R&D systems	MAB4068	Proteomic
cd160	R&D systems	MAB6700	Proteomic
AFP	R&D systems	MAB1368	Proteomic
CXCL12(SDF1)	R&D systems	MAB350	Proteomic
CSF3(GCSF)	R&D systems	MAB214	Proteomic
S100A7/Psoriasin	n/a	n/a (clone: 396)	Proteomic
TP73	Santa Cruz Biotechnology	sc-56190	Proteomic

HIST1H1D	Abcam	ab24174	Proteomic
THBS1	LSBio	LS-B2570	Proteomic
EPHA2	Santa Cruz Biotechnology	sc-924	Proteomic
EGFR	Santa Cruz Biotechnology	sc-003	Proteomic
RAF1	Santa Cruz Biotechnology	sc-7267	Proteomic
HGFAC	Santa Cruz Biotechnology	sc-7949	Proteomic
CDKN2B	Santa Cruz Biotechnology	sc-613 (lot C0410)	Proteomic
LAMA1	Santa Cruz Biotechnology	sc-5582	Proteomic
Cdkn2a	Santa Cruz Biotechnology	sc-32749	Proteomic
PGK1	Santa Cruz Biotechnology	sc-48342	Proteomic
IL13RA1	SDI	2505.00.02	Proteomic
DDB2	SDI	2555.00.02	Proteomic
BRCA1	SDI	4099.00.02	Proteomic
TGFB2	SDI	2441.00.02	Proteomic
COL5A3	Santa Cruz Biotechnology	sc-240246	Proteomic
PLG	SDI	2640.00.02	Proteomic
BRD2	SDI	2799.00.02	Proteomic
BIRC2	SDI	2969.00.02	Proteomic
CD70	SDI	2651.00.02	Proteomic
ABCA3	SDI	3828.00.02	Proteomic
GPR19	SDI	2676.00.02	Proteomic
LST1	SDI	3011.00.02	Proteomic
GADD45G	SDI	2966.00.02	Proteomic
INHBA	SDI	3343.00.02	Proteomic
CAMK1	SDI	3120.00.02	Proteomic
ATG7	SDI	3552.00.02	Proteomic
ZMIZ1	SDI	3719.00.02	Proteomic
BCL3	Santa Cruz Biotechnology	sc-13038	Proteomic
SOCS7	SDI	2192.00.02	Proteomic
TRPV6	Aviva	OACD01210	Proteomic
NTNG2	ProteinTech	12910-2-AP	Proteomic
NTNG2	Sigma	HPA071290	Proteomic
TRPV6	Novus	NBP2-32372	Proteomic
EVI1	SDI	2810.00.02	Glycomic
SIGLEC6	Aviva	ARP41925_P050	Glycomic
BMF	Sigma	B1684-100ug	Glycomic
TFF3	R&D systems	MAB4408	Glycomic
TFF3	Aviva	OACD07660	Glycomic
WNT5B	SDI	2301.00.02	Glycomic
ALPL	SDI	3292.00.02	Glycomic
PHB	SDI	2684.00.02	Glycomic

VHL	SDI	2319.00.02	Glycomic
BSG	SDI	2602.00.02	Glycomic
JMJD1C	SDI	2983.00.02	Glycomic
JMJD1C	Abnova	H00221037-M03	Glycomic
JMJD1C	Novus	NBP1-77072	Glycomic
FETUB	R&D systems	MAB17251	Glycomic
C4B	Abcam	AB54902	Glycomic
AFP	SDI	2544.00.02	Glycomic
TET1	SDI	2126.00.02	Glycomic
ILK	Sigma	I1907	Glycomic
ILK	R&D systems	443208	Glycomic
ILK	ProteinTech	12955-I-AP	Glycomic
TNFAIP6	R&D systems	MAB2104	Glycomic
CD22	PeproTech	500-P227	Glycomic
COL24A1	Abcam	AB57249	Glycomic
COL24A1	Abnova	H00255631-M07	Glycomic
CD44	SDI	4084.00.02	Glycomic
SUFU	SDI	2487.00.02	Glycomic
HP	Sigma	H8636-1vl	Glycomic
SCN3B	SDI	3747.00.02	Glycomic
ALDOC	Aviva	ARP48274_P050	Glycomic
EN1	SDI	2104.00.02	Glycomic
GALK1	Abnova	H00002584-M01	Glycomic
TNFRSF8	SDI	3490.00.02	Glycomic
KDM5B	SDI	2226.00.02	Glycomic
SLC30A8	Origene	TA590368	Glycomic
PW-1L (CCSP-1)	CCSP-1 Antibody	PW-1L	Glycomic
THBS1	LSBio	LS-B2570	Glycomic
EDN1	Abnova	H00001906-M01	Glycomic
KRT10	Abnova	H00003858-M01	Glycomic
STIP1	Abcam	AB37752	Glycomic
CASP8	SDI	2802.00.02	Glycomic
VWF	Sigma	F3520	Glycomic
MUC17	AbD Serotec	MCA1837	Glycomic
MUC5AC	Abnova	H00004586-M07	Glycomic
MUC16	SDI	2545.00.02	Glycomic
MUC6	Novus	46760002	Glycomic
MUC16 (CA125)	Abcam	AB81871	Glycomic
MUC1	Abcam	AB15481	Glycomic

Table S2. Distribution of CT parameters in FH cohort 1 and 2.

Variable	FH1 (N = 135)	FH2 (N = 149)
Contrast enhancement (yes)	56 (41.5)	36 (24.2)
kVp		
80	1(0.7)	1 (0.7)
100	13 (9.6)	8 (5.4)
110	1 (0.7)	0 (0.0)
120	117 (86.7)	136 (91.3)
130	2 (1.5)	3 (2.0)
140	1 (0.7)	1 (0.7)
Scan modality		
GE	96 (71.1)	123 (82.6)
PHILIPS	7 (5.2)	2 (1.3)
SIEMENS	23 (17.0)	17 (11.4)
TOSHIBA	9 (6.7)	7 (4.7)
Slice thickness (mm)		
1	3 (2.2)	0 (0.0)
1.25	9 (6.7)	4 (2.7)
1.5	1 (0.7)	0 (0.0)
2	12 (8.9)	8 (5.4)
2.5	65 (48.1)	94 (63.1)
3	12 (8.9)	9 (6.0)
3.75	3 (2.2)	2 (1.3)
4	1 (0.7)	0 (0.0)
5	29 (21.5)	31 (20.8)
10	0 (0)	1 (0.7)

Table S3. Selected 94 reproducible radiomics features input into Lasso selection. * All highly skewed (|skew| > 1) and strictly positive radiomic features were log-transformed to reduce skewness and increase information content.

Radiomics features	Category
original_shape_Flatness	Shape feature
original_shape_LeastAxisLength	Shape feature
original_firstorder_Uniformity *	First-order feature
original_glcm_Idmn*	Texture feature
original_glcm_Imc1	Texture feature
original_glcm_SumAverage	Texture feature
original_gldm_LowGrayLevelEmphasis*	Texture feature
original_glszm_ZonePercentage	Texture feature

original_ngtdm_Busyness*	Texture feature
wavelet-LLH_firstorder_Entropy	Wavelet feature
wavelet-LLH_glcm_Idm	Wavelet feature
wavelet-LLH_glcm_Imc1	Wavelet feature
wavelet-LLH_glcm_Imc2	Wavelet feature
wavelet-LLH_glcm_JointEntropy	Wavelet feature
wavelet-LLH_glcm_MaximumProbability	Wavelet feature
wavelet-LLH_gldm_DependenceNonUniformityNormalized*	Wavelet feature
wavelet-LLH_gldm_DependenceVariance	Wavelet feature
wavelet-LLH_gldm_LargeDependenceEmphasis	Wavelet feature
wavelet-LLH_glrlm_GrayLevelNonUniformityNormalized	Wavelet feature
wavelet-LLH_glrlm_LongRunEmphasis*	Wavelet feature
wavelet-LLH_glrlm_RunEntropy*	Wavelet feature
wavelet-LLH_glrlm_RunLengthNonUniformity*	Wavelet feature
wavelet-LLH_glrlm_RunPercentage	Wavelet feature
wavelet-LLH_glrlm_RunVariance	Wavelet feature
wavelet-LLH_glszm_ZonePercentage*	Wavelet feature
wavelet-LHL_firstorder_Median	Wavelet feature
wavelet-LHL_glcm_Idn*	Wavelet feature
wavelet-LHL_glcm_Imc1	Wavelet feature
wavelet-LHL_glcm_Imc2	Wavelet feature
wavelet-LHL_gldm_DependenceEntropy*	Wavelet feature
wavelet-LHL_gldm_SmallDependenceLowGrayLevelEmphasis*	Wavelet feature
wavelet-LHL_glszm_ZoneEntropy*	Wavelet feature
wavelet-LHH_glcm_Imc1	Wavelet feature
wavelet-LHH_glcm_Imc2*	Wavelet feature
wavelet-LHH_gldm_SmallDependenceEmphasis*	Wavelet feature
wavelet-LHH_glrlm_GrayLevelNonUniformityNormalized	Wavelet feature
wavelet-LHH_glrlm_RunLengthNonUniformityNormalized*	Wavelet feature
wavelet-HLL_firstorder_Mean	Wavelet feature
wavelet-HLL_glcm_ClusterTendency*	Wavelet feature
wavelet-HLL_glcm_Idmn*	Wavelet feature
wavelet-HLL_glcm_Idn*	Wavelet feature
wavelet-HLL_glcm_Imc1	Wavelet feature
wavelet-HLL_glcm_Imc2	Wavelet feature
wavelet-HLL_gldm_DependenceNonUniformityNormalized*	Wavelet feature
wavelet-HLL_gldm_SmallDependenceEmphasis*	Wavelet feature
wavelet-HLL_ngtdm_Busyness*	Wavelet feature
wavelet-HLH_firstorder_10Percentile	Wavelet feature

wavelet-HLH_firstorder_90Percentile	Wavelet feature
wavelet-HLH_firstorder_Entropy*	Wavelet feature
wavelet-HLH_firstorder_InterquartileRange*	Wavelet feature
wavelet-HLH_firstorder_MeanAbsoluteDeviation*	Wavelet feature
wavelet-HLH_firstorder_Uniformity*	Wavelet feature
wavelet-HLH_glcm_DifferenceAverage*	Wavelet feature
wavelet-HLH_glcm_Idm*	Wavelet feature
wavelet-HLH_glcm_Imc2*	Wavelet feature
wavelet-HLH_glcm_JointEnergy	Wavelet feature
wavelet-HLH_glcm_SumEntropy*	Wavelet feature
wavelet-HLH_gldm_DependenceEntropy*	Wavelet feature
wavelet-HLH_gldm_DependenceNonUniformityNormalized*	Wavelet feature
wavelet-HLH_gldm_DependenceVariance	Wavelet feature
wavelet-HLH_gldm_LargeDependenceEmphasis	Wavelet feature
wavelet-HLH_glrlm_GrayLevelNonUniformityNormalized	Wavelet feature
wavelet-HLH_glrlm_RunPercentage*	Wavelet feature
wavelet-HLH_glrlm_RunVariance	Wavelet feature
wavelet-HLH_ngtdm_Busyness*	Wavelet feature
wavelet-HHH_gldm_DependenceVariance	Wavelet feature
wavelet-HHH_gldm_LargeDependenceEmphasis*	Wavelet feature
wavelet-LLL_firstorder_90Percentile	Wavelet feature
wavelet-LLL_firstorder_Energy*	Wavelet feature
wavelet-LLL_firstorder_Entropy	Wavelet feature
wavelet-LLL_firstorder_Kurtosis*	Wavelet feature
wavelet-LLL_firstorder_Skewness	Wavelet feature
wavelet-LLL_glcm_ClusterProminence*	Wavelet feature
wavelet-LLL_glcm_ClusterShade	Wavelet feature
wavelet-LLL_glcm_DifferenceAverage*	Wavelet feature
wavelet-LLL_glcm_DifferenceEntropy	Wavelet feature
wavelet-LLL_glcm_DifferenceVariance*	Wavelet feature
wavelet-LLL_glcm_Idn	Wavelet feature
wavelet-LLL_glcm_Imc1	Wavelet feature
wavelet-LLL_glcm_InverseVariance	Wavelet feature
wavelet-LLL_glcm_JointEnergy*	Wavelet feature
wavelet-LLL_glcm_JointEntropy	Wavelet feature
wavelet-LLL_glcm_SumEntropy*	Wavelet feature
wavelet-LLL_gldm_DependenceNonUniformityNormalized	Wavelet feature
wavelet-LLL_glrlm_GrayLevelNonUniformity*	Wavelet feature
wavelet-LLL_glrlm_GrayLevelNonUniformityNormalized*	Wavelet feature

wavelet-LLL_glrlm_LongRunHighGrayLevelEmphasis*	Wavelet feature
wavelet-LLL_glrlm_RunLengthNonUniformityNormalized*	Wavelet feature
wavelet-LLL_glrlm_ShortRunHighGrayLevelEmphasis*	Wavelet feature
wavelet-LLL_glszm_SmallAreaEmphasis	Wavelet feature
wavelet-LLL_glszm_ZonePercentage	Wavelet feature
wavelet-LLL_ngtdm_Busyness*	Wavelet feature
wavelet-LLL_ngtdm_Coarseness*	Wavelet feature
wavelet-LLL_ngtdm_Contrast*	Wavelet feature

Table S4. Comparison of demographics between FH cohort 1 and 2. Body mass index (BMI), Non-small cell lung carcinoma (NSCLC), Adenocarcinoma (AD), squamous cell carcinoma (SCC). * Missing values present in BMI (n=1), BMI class (n=1), Family History of lung cancer (n=3), Years since quitting smoking (n=2). † Fisher's exact test (categorical) or Wilcoxon rank-sum test (continuous variables) comparing patients with and without malignancy or two FH cohorts.

		**FH Cohort***		
**Variable / Level**	**Overall (N = 284)**	**1 (n = 135)**	**2 (n = 149)**	**P†**
Gender (M)	149 (52.5%)	64 (47.4%)	85 (57.0%)	0.12
Age	66.50 (33.00 - 94.00)	67.00 (40.0.0 - 91.00)	66.00 (33.00 - 94.00)	0.54
Race				0.77
African American	6 (2.1%)	1 (0.7%)	5 (3.4%)	
Asian	13 (4.6%)	5 (3.7%)	8 (5.4%)	
Caucasian	255 (89.8%)	125(92.6%)	130(87.2%)	
Hispanic or Latino	2 (0.7%)	1(0.7%)	1 (0.7%)	
Native American	3 (1.1%)	1 (0.7%)	2 (1.3%)	
Native Hawaiian/Pacific Islander	2 (0.7%)	1 (0.7%)	1 (0.7%)	
Unknown or Not Reported	3 (1.1%)	1 (0.7%)	2 (1.3%)	
BMI*	26.60 (16.14 - 58.65)	26.70 (17.85 - 58.65)	26.40 (16.14 - 52.93)	0.69
BMI.class*				0.90
normal	113 (39.9%)	52 (38.8%)	61 (40.9%)	
overweight	93 (32.9%)	44 (32.8%)	49 (32.9%)	
obese	77 (27.2%)	38 (28.4%)	39 (26.2%)	
Smoking Status				**0.006**
Current smoker	74 (26.1%)	45 (33.3%)	29 (19.5%)	
Former smoker	142 (50.0%)	67 (49.6%)	75 (50.3%)	
Never smoker	68 (23.9%)	23 (17.0%)	45(30.2%)	
Pack Years	25 (0 - 183)	28.5 (0 - 183)	22.5 (0 - 180)	0.06
Prior Cancer History (yes)	80 (28.2%)	47 (34.8%)	33 (22.1%)	**0.02**
Family History of lung cancer* (yes)	77 (27.4%)	35 (26.1%)	42 (28.6%)	0.69
Years since quitting smoking*	0 (0 - 64)	0 (0 - 58)	1 (0 - 64)	0.19
Malignancy (yes)	140 (49.3%)	69 (51.1%)	71 (47.7%)	0.63
Histology for NSCLC				0.21
AD	107 (76.4%)	56(81.2%)	51 (71.8%)	

AD, SCC	1 (0.7%)	0 (0.0%)	1 (1.4%)	
Large cell carcinoma	1 (0.7%)	1 (1.4%)	0 (0.0%)	
SCC	31 (22.1%)	12 (17.4%)	19 (26.8%)	
NSCLC stage				**0.008**
0	2 (1.4%)	2 (2.9%)	0 (0.0%)	
I	95 (67.9%)	40 (58.0%)	55 (77.5%)	
II	12 (8.6%)	4 (5.8%)	8 (11.3%)	
III	13 (9.3%)	9 (13.0%)	4 (5.6%)	
IV	18 (12.9%)	14 (20.3%)	4 (5.6%)	

Table S5. Distribution of semantic features in Fred Hutch (FH) 1 and FH2 cohorts.

Variable	FH1 (n=135)			FH2 (n=149)		
	Control (n = 66)	Case (n = 69)	P†	Control (n = 78)	Case (n = 71)	P†
Nodule location						
Left upper lobe	14 (21.2)	16 (23.2)	0.84	10 (12.8)	16 (22.5)	0.13
Lingula	2 (3.0)	0 (0.0)	0.24	5 (6.4)	3 (4.2)	0.72
Left lower lobe	9 (13.6)	4 (5.8)	0.15	12 (15.4)	15 (21.1)	0.40
Right upper lobe	17 (25.8)	29 (42.0)	0.07	18 (23.1)	23 (32.4)	0.27
Right middle lobe	7 (10.6)	1 (1.4)	**0.03**	7 (9.0)	3 (4.2)	0.33
Right lower lobe	17 (25.8)	19 (27.5)	0.85	25 (32.1)	11 (15.5)	**0.02**
Other	0 (0.0)	0 (0.0)	NA	1 (1.3)	0 (0.0)	>0.99
Vector Magnitude [mm]	20.0 (9.0 - 49.0)	31.0 (13.0 - 61.0)	**<0.001**	20.0 (9.0 - 57.0)	31.0 (11.0 - 60.0)	**<0.001**
Long axis of solid component [mm]	NA	10.0 (3.0 - 30.0)	NA	7.0 (4.0 - 13.0)	10.0 (3.0 - 28.0)	0.24
Nodule margin						
Smooth	25 (37.9)	1 (1.4)	**<0.001**	34 (43.6)	2 (2.8)	**<0.001**
Irregular	33 (50.0)	51 (73.9)	**<0.01**	39 (50.0)	43 (60.6)	0.25
Scalloped	8 (12.1)	15 (21.7)	0.17	5 (6.4)	23 (32.4)	**<0.001**
Spiculated	1 (1.5)	18 (26.1)	**<0.001**	0 (0.0)	14 (19.7)	**<0.001**
Corona.radiata	0 (0.0)	9 (13.0)	**<0.01**	0 (0.0)	3 (4.2)	0.11
Nodule density						
Solid	63 (95.5)	48 (69.6)	**<0.001**	68 (87.2)	47 (66.2)	**<0.001**
Part-solid	0 (0.0)	19 (27.5)	**<0.001**	5 (6.4)	24 (33.8)	**<0.001**
Non-solid	3 (4.5)	2 (2.9)	0.68	5 (6.4)	0 (0.0)	0.06
Cavitary	3 (4.5)	4 (5.8)	>0.99	3 (3.8)	2 (2.8)	>0.99
Pseudocavitary	1 (1.5)	2 (2.9)	>0.99	0 (0.0)	3 (4.2)	0.11
Calcification with benign pattern	1 (1.5)	0 (0.0)	0.49	2 (2.6)	0 (0.0)	0.50
Calcification with non-benign.pattern	1 (1.5)	0 (0.0)	0.49	0 (0.0)	0 (0.0)	NA
Number of nodules						
1 nodule	11 (16.7)	14 (20.3)	0.66	18 (23.1)	14 (19.7)	0.69
2-6 nodules	41 (62.1)	42 (60.9)	>0.99	42 (53.8)	45 (63.4)	0.25
More than 6 nodules	14 (21.2)	13 (18.8)	0.83	18 (23.1)	12 (16.9)	0.42

Emphysema score	0.8 (0.5 - 4.0)	1.0 (0.5 - 4.0)	0.02	0.5 (0.5 - 3.0)	1.0 (0.5 - 3.0)	<0.001
0 (none affected)	26 (39.4)	18 (26.1)		46 (59.0)	22 (31.0)	
0.5 (0~5% affected)	20 (39.4)	14 (20.3)		17 (21.8)	18 (25.4)	
1.0 (6~25% affected)	9 (13.6)	19 (27.5)		5 (6.4)	16 (22.5)	
2.0 (26~50% affected)	4 (6.1)	7 (10.1)		4 (5.1)	9 (12.7)	
3.0 (51~75% affected)	4 (6.1)	9 (13.0)		6 (7.7)	6 (8.5)	
4.0 (>75% affected)	3 (4.5)	2 (2.9)		0 (0.0)	0 (0.0)	
Pattern of emphysema						
Centrilobular	36 (54.5)	51 (73.8)	0.03	29 (37.1)	46 (64.8)	0.68
Panlobular	4 (6.0)	5 (7.2)	>0.99	4 (5.1)	1 (1.4)	0.08
Paraseptal	4 (6.0)	5 (7.2)	>0.99	7 (9.0)	11 (15.5)	>0.99
Region of emphysema						
Upper lobe predominant	32 (48.5)	43 (62.3)	0.59	29 (37.2)	46 (64.8)	0.68
Lower lobe predominant	1 (1.5)	0 (0.0)	0.44	0 (0.0)	1 (1.4)	>0.99
Diffuse	7 (10.6)	7 (10.1)	0.77	3 (3.8)	2 (2.8)	0.38
Other	0 (0.0)	1 (1.4)	>0.99	0 (0.0)	0 (0.0)	>0.99
Evidence of fibrosis	3 (4.5)	5 (7.2)	0.72	8 (10.3)	7 (9.9)	>0.99
Evidence of asbestosis	0 (0.0)	0 (0.0)	NA	1 (1.3)	0 (0.0)	>0.99
Evidence of invasion						
No invasion	63 (95.5)	57 (82.6)	0.03	77 (98.7)	53 (74.6)	<0.001
Invasion into pleura or fissure	2 (3.0)	9 (13.0)	0.06	1 (1.3)	16 (22.5)	<0.001
Invasion into other structures like hilum, mediastinum, large airway, heart or great vessels, chest wall or vertebrae	1 (1.5)	3 (4.3)	0.62	0 (0.0)	2 (2.8)	0.23
Lymph nodes						
No lymphadenopathy	46 (69.7)	33 (47.8)	0.01	39 (50.0)	40 (56.3)	0.51
No pathologic enlargement	7 (10.6)	8 (11.6)	>0.99	31 (39.7)	18 (25.4)	0.08
Non-pathologic calcified node	2 (3.0)	1 (1.4)	0.61	2 (2.6)	2 (2.8)	>0.99
Enlargement of ipsilateral intrapulmonary, peribronchial or hilar	1 (1.5)	3 (4.3)	0.62	1 (1.3)	1 (1.4)	>0.99
Enlargement of ipslateral mediastinal, subcarinal nodes	3 (4.5)	7 (10.1)	0.33	2 (2.6)	5 (7.0)	0.26
Enlargement of contralateral mediastinal or hilar	7 (10.6)	17 (24.6)	0.04	3 (3.8)	5 (7.0)	0.48

Table S6. Plasma biomarker input into Lasso selection.

		FH1 Cohort		FH2 Cohort	
Biomarker type	Gene Name	coef	pvalue	coef	pvalue
SleA	VHL	0.218	0.101	0.642	0.001
SleA	WNT5B	1.052	0.000	0.429	0.007
SleA	TNFAIP6	0.224	0.106	0.383	0.017
SleA	FETUB	0.261	0.050	0.299	0.039

SleA	CD22	0.614	0.004	0.248	0.075
SleA	MUC16	0.463	0.003	0.246	0.082
SleA	SCN3B	0.329	0.030	0.260	0.087
SleX	TET1	0.473	0.002	0.478	0.004
SleX	FETUB	0.261	0.050	0.380	0.009
SleX	THBS1	0.399	0.009	0.410	0.021
SleX	WNT5B	1.052	0.000	0.333	0.028
SleX	C4B	0.259	0.063	0.356	0.029
SleX	EVI1	0.358	0.040	0.324	0.029
SleX	PHB	0.405	0.014	0.335	0.030
SleX	AFP	0.496	0.005	0.296	0.045
SleX	ALPL	0.710	0.000	0.274	0.053
SleX	HP	0.253	0.089	0.304	0.059
SleX	TNFRSF8	0.767	0.000	0.244	0.073
SleX	KDM5B	0.655	0.000	0.240	0.087
IgG	SMARCA2	0.234	0.081	0.454	0.004
IgG	RGL1	0.722	0.000	0.469	0.011
IgG	MDH2	0.450	0.003	0.390	0.012
IgG	NRP1	0.301	0.045	0.594	0.014
IgG	CLNS1A	0.405	0.022	0.360	0.026
IgG	WNT5B	0.296	0.036	0.325	0.043
IgG	YES1	0.214	0.093	0.311	0.049
IgG	WNT10A	0.613	0.000	0.261	0.079
IgG	CDC25C	0.420	0.009	0.253	0.080
IgG	RECQL4	0.406	0.014	0.249	0.093
IgM	WNT10A	0.540	0.004	0.475	0.004
IgM	TRPV6	0.343	0.034	0.217	0.082
IgM	EPHA2	0.370	0.037	0.203	0.093
Proteomic	IL13RA1	0.477	0.001	0.559	0.002
Proteomic	TRPV6	0.348	0.038	0.533	0.003
Proteomic	FMN1	0.393	0.018	0.481	0.006
Proteomic	SPFH2	0.377	0.028	0.477	0.006
Proteomic	EXT2	0.226	0.092	0.370	0.009
Proteomic	NCK1	0.403	0.010	0.428	0.009
Proteomic	PHLPPL	0.431	0.005	0.498	0.012
Proteomic	CAMK1	0.309	0.056	0.407	0.018
Proteomic	SMAD2	0.405	0.012	0.347	0.021
Proteomic	NTNG2	0.241	0.087	0.374	0.029
Proteomic	BIRC2	0.258	0.085	0.315	0.032
Proteomic	SMAD2	0.405	0.012	0.352	0.035
Proteomic	CSF3	0.257	0.066	0.330	0.040
Proteomic	ERCC2	0.486	0.004	0.339	0.041
Proteomic	KIFC3	0.361	0.039	0.265	0.051
Proteomic	INHBC	0.553	0.002	0.314	0.052
Proteomic	SLC30A8	0.345	0.032	0.264	0.056
Proteomic	LIFR	0.471	0.042	0.269	0.060

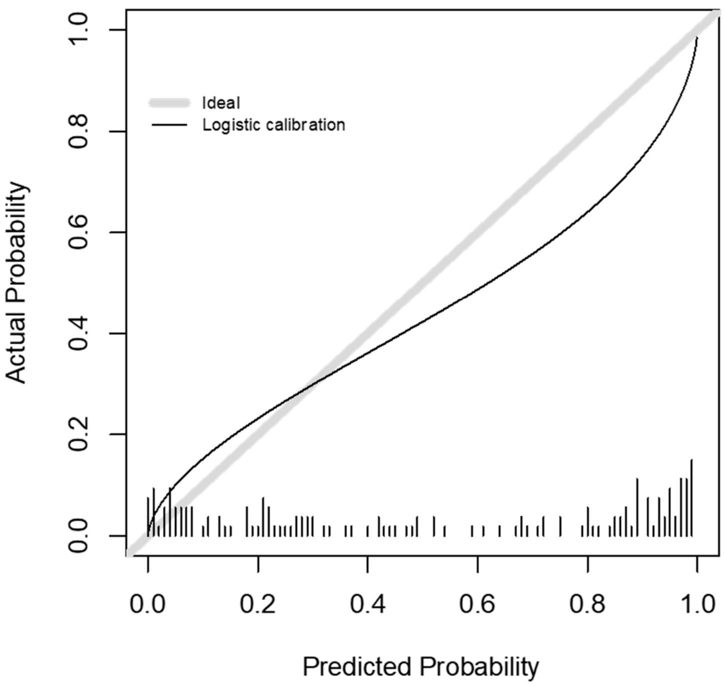

Figure S1. Calibration of the PSR model in FH2 cohort. The fitted logistic calibration curve has intercept -0.31 (95% CI: -0.73, 0.096) and slope 0.64 (95% CI: 0.45, 0.86). An ideal calibration curve has intercept of zero and slope of one. These estimates of slope and intercept suggest an adequate overall calibration, although as seen by the curve the model may overestimate high risk and, to a lesser extent, underestimate low risk.

65. Zhao, B.; Shwartz, L.H.; Kris, M.G. Data from RIDER_Lung CT. The Cancer Imaging Archive. **2015**. https://wiki.cancerimagingarchive.net/display/Public/RIDER+Lung+CT.
66. Wee, L.; Aerts, H.J.L.; Kalendralis, P.; Dekker, A. Data from NSCLC-Radiomics-Interobserver1 [Data set]. The Cancer Imaging Archive. **2019**. https://wiki.cancerimagingarchive.net/display/Public/NSCLC-Radiomics-Interobserver1
67. Du, D.; Gu, J.; Chen, X.; Lv, W.; Feng, Q.; Rahmim, A.; Wu, H.; Lu, L. Integration of PET/CT Radiomics and Semantic Features for Differentiation between Active Pulmonary Tuberculosis and Lung Cancer. *Mol Imaging Biol* **23**, 287–298 (2021).
